# Can returning home for entrepreneurship improve the agricultural economic resilience: Investigation based on the pilot policy of returning home for entrepreneurship

**DOI:** 10.1371/journal.pone.0342232

**Published:** 2026-03-30

**Authors:** Xiaowen Yu, Hongyu Ma

**Affiliations:** School of Management, South Campus, Northwest A&F University School of Economics and Management, Xianyang, China; Western Carolina University, UNITED STATES OF AMERICA

## Abstract

Against the backdrop of the ongoing transition of rural China from a “labor migrant economy” to an “entrepreneurial economy”, this study utilizes panel data covering 2005 counties nationwide in China during the period 2012–2022. Employing a Difference-in-Differences (DID) model integrated with a mediating effect approach, this study comprehensively examines the impact of the pilot hometown-return entrepreneurship policy on agricultural economic resilience and its intrinsic mechanisms of action. The results show that agricultural economic resilience exhibits a steady upward trend and significant spatial disparity, characterized by a spatial distribution pattern of “high in the east, low in the west, high in the north, and low in the south”. The pilot hometown-return entrepreneurship policy exerts a significantly positive effect on enhancing agricultural economic resilience, with this positive impact being more pronounced in western China and less substantial in eastern China. Mechanism analysis reveals that increasing farmers’ income and advancing the rationalization of the industrial structure are the key transmission channels through which the pilot hometown-return entrepreneurship policy enhances agricultural economic resilience. These findings contribute to a better understanding of the extent to which and the mechanisms through which the pilot hometown-return entrepreneurship policy enhances agricultural economic resilience. It is imperative for all regions to continuously strengthen policy support and guidance for returning farmers and enhance regional attractiveness in light of local conditions, thereby fostering the high-quality development of the agricultural economy.

## 1 Introduction

With the intensification of global climate change and market volatility, agricultural economic resilience (AER) has become a critical issue in safeguarding food security and achieving sustainable development [1]. Currently, China’s agricultural development is confronted with multiple challenges: frequent natural disasters undermine the stability of the production side, volatility in international food prices exacerbates the transmission of market risks, and the continuous outflow of rural labor leads to factor misallocation. These issues jeopardize the stability of the food supply chain and hinder the endogenous growth momentum of the rural economy. Against this backdrop, it is imperative to optimize the reconfiguration of the agricultural production system through policy tools and enhance the system’s resilience to external shocks. This has emerged as a pivotal proposition for the transition towards agricultural modernization.

In recent years, farmers returning to their hometowns to start businesses, an important practical approach to rural revitalization, have gradually demonstrated unique value in resource integration and industrial upgrading. By introducing modern technologies, advanced management experience, and market information, returning entrepreneurs drive the transformation of traditional agriculture toward a diversified and high-value-added model, injecting new momentum to invigorate the rural economy [2]. At the policy level of exploring pilot initiatives, pilot policies promote the two-way flow of urban-rural factors through financial support, platform construction, and skills training, thereby addressing rural resource mismatch issues in rural development [3]. What is the intrinsic link between this entrepreneurial practice model and the enhancement of AER? Is this model universally applicable? Answering these questions is not only crucial to the scientificity and precision of policy design but also of far-reaching significance for constructing a sustainable agricultural economic system.

Agricultural economic resilience (AER), a core concept denoting the ability of agricultural systems to cope with shocks and achieve recovery, has attracted extensive attention from the academic community in recent years. Bullock et al. defined AER as “the ability to maintain adequate production of nutritious food under environmental perturbations”, emphasizing its multidimensional spatial scale characteristics that cover farmland, farm, regional, and global levels [4]. In addition, Erika constructed a four-dimensional indicator system comprising financial flexibility, development path stability, industrial diversification, and export market diversification from the perspective of economic resilience [5]. Zhang conceptualized AER as a dynamic “resistance-adaptation-transformation” process in the context of major grain-producing regions in Northeast China [6]. In a study examining influencing factors, Ikien found that direct payments under the EU Common Agricultural Policy (CAP) enhance AER by improving agricultural financial capacity [7]. However, excessive reliance on subsidies could undermine market adaptability. Zuo found that rural industrial integration enhances AER in both eastern and central China, with AER levels in these regions being 1.5-fold and 1.3-fold higher than the national average, respectively [8]. Several factors drive these improvements, including extending industrial chain value, optimizing factor allocation efficiency, and enhancing risk diversification capacity. Li reported that the spillover effects of industrial agglomeration are also substantial [9]. Notably, promoting industrial agglomeration, strengthening social services, and improving farmers’ production efficiency are three important approaches to enhancing the agricultural economic resilience index. Yang and Zhang’s empirical study revealed that promoting sustainable agriculture can significantly enhance farm-level resilience, with the strongest positive impact on employee well-being and safety, as well as ecological rights protection measures [10]. Furthermore, Ma identified a threshold effect of financial support policies on AER enhancement, with marginal benefits starting to decline when financial subsidies account for more than 3.5% of agricultural output [11].

As an emerging research area, the evaluation of the pilot hometown-return entrepreneurship policy effect using the Difference-in-Differences (DID) method exhibits multidimensional characteristics. In the early stages of research, Hooks demonstrated through empirical studies that entrepreneurial resilience, as a mediating variable, significantly enhances the entrepreneurial intention of returning entrepreneurs by improving opportunity perception, which provides a new perspective for understanding the policy’s mechanism of action [12]. Sun and Sun employed 35 software parks of the National Torch Plan Software Industry Base as research samples and found that the agglomeration of returning entrepreneurs positively impacts regional agricultural total factor productivity [13]. Zhang evaluated the effect of the pilot policy implemented in 341 counties by the Chinese government since 2015, finding that the construction of entrepreneurial platforms, financial support, and skills training significantly promote the return of production factors [10]. However, there are significant regional differences in policy effectiveness. For example, among China’s eastern, central, and western regions, the southwest region (a sub-region of western China) exhibits a synergistic advantage in policy implementation due to the proximity effect. In contrast, the industrial crowding-out effect in the eastern coastal region reduces policy responsiveness by 23% [14]. Furthermore, optimizing the mix of entrepreneurial policy tools is particularly critical. Adesoji found that a three-dimensional support system comprising tax incentives, infrastructure improvements, and cross-border trade training can increase the survival rate of entrepreneurial firms from 43% to 67% [15].

Although existing studies have made significant progress in the conceptual framework, measurement methods, and influencing factors of agricultural economic resilience (AER), AER encompasses not only the buffering capacity to withstand short-term shocks but also long-term adaptability and economic recovery efficiency. Its enhancement requires breaking through the limitations of a single production dimension. By activating local resources and optimizing factor allocation, hometown-return entrepreneurship may serve as an important bridge linking the vitality of micro-subjects with macro-policy objectives. However, systematic research remains scarce on how the Difference-in-Differences (DID) method reveals the role of the pilot hometown-return entrepreneurship policy in the evolution of AER. Similarly, while existing studies focus on the employment-driven effect of hometown-return entrepreneurship, they fail to clarify its transmission path. In practice, the appropriateness of policy tools, differences in regional development bases, and market demand orientations may influence the ultimate policy effect. How to balance policy intervention and market mechanisms in practice and establish a multi-stakeholder collaborative resilience cultivation mechanism is a topic that requires in-depth exploration.

This study possesses innovative value in the following three aspects: First, by constructing a multidimensional panel database covering county-level administrative units nationwide and leveraging a larger sample size and more granular data units, the robustness of the empirical analysis has been effectively enhanced. Second, the core indicator of agricultural economic resilience (AER) has been innovatively incorporated to establish a novel evaluation framework for the effectiveness of the pilot hometown-return entrepreneurship policy based on the Difference-in-Differences (DID) method. Third, this study not only quantitatively measures the direct impact of the pilot hometown-return entrepreneurship policy on AER but also systematically identifies two core transmission mechanisms—farmers’ income level and industrial rationalization level—thereby providing multidimensional decision support for optimizing the rural revitalization policy system.

The paper is structured as follows. Section 2 expounds on theoretical analysis and formulates research hypotheses. Section 3 details Materials and Methods. Section 4 and Section 5 respectively present the results and conduct discussions. Section 6 summarizes the conclusion.

## 2 Policy background and theoretical analysis

### 2.1 Policy background

To more effectively support and guide rural migrant workers in returning to their hometowns to start businesses, ten ministries and commissions, including the National Development and Reform Commission and the Ministry of Agriculture and Rural Affairs of the People’s Republic of China, jointly approved the establishment of 90, 116, and 135 pilot zones for returnee entrepreneurship in February 2016, December 2016, and October 2017, respectively. By strengthening the integration of park resources and the development of service platforms, and optimizing policy support, project guidance, and channel connectivity, these authorities have continuously removed various barriers to entrepreneurship, facilitated the inflow of material and human capital into the pilot zones, and boosted their endogenous development momentum.

According to the requirements of relevant application documents, the development and reform commissions of each province, autonomous region, and municipality directly under the central government will organize relevant counties (cities, districts) to prepare materials for independent application in accordance with relevant requirements. They will then work with relevant departments to study and determine the recommended list of pilot areas. Finally, the national development and reform commission will determine and publish the list of pilot areas, and periodically organize relevant departments to conduct policy performance evaluations, employment monitoring, and supervision assessments of pilot areas. The policy support and guidance from governments at all levels have greatly promoted the return of migrant farmers to their hometowns for entrepreneurship. Among them, in 2020 alone, the number of entrepreneurs returning to their hometowns in China reached 10.1 million, with an average of 6.3 people being able to find stable employment and 17.3 people being able to find flexible employment per entrepreneurial project, demonstrating a strong ability to drive employment.

**Fig 1:**
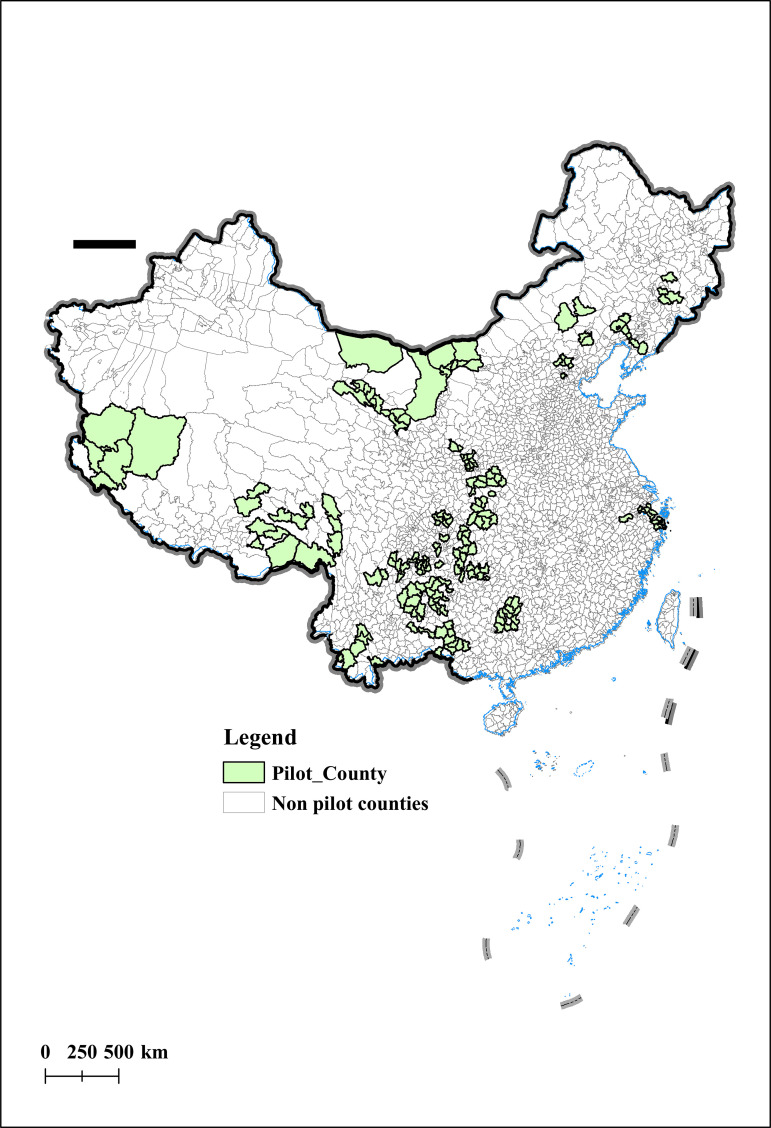
Sample of Counties for Returning Home to Start a Business. Notes: The vector maps was derived from publicly available data from the National Catalogue Service of Geographic Information in China (GS(2023)2765).

This event is planned and promoted by the national government, greatly eliminating the interference of local governments in the selection results, providing a good quasi natural experiment for this article to effectively identify the impact of rural entrepreneurship on agricultural economic resilience. Finally, 243 counties selected as “pilot counties for returning home entrepreneurship” in the research sample were selected to form the experimental group of this article. Fig 1 shows the geographic spatial distribution of the pilot counties for returning home entrepreneurship involved in this study. As shown in the figure, the pilot counties for returning home to start businesses present a geographical pattern of more in the west and less in the east, with dense areas in the south and sparse areas in the north. It is worth noting that the concentration trend of pilot counties shows a northeast southwest direction, which highly overlaps with the population density line.

### 2.2 Theoretical implications of Agricultural Economic Resilience (AER)

Agricultural economic systems inherently possess inherent risks and uncertainties. Some scholars argue that risk management can enhance the identification and prevention of specific risks. However, due to the unpredictable nature of uncertainty shocks, improving a system’s resilience cannot be confined solely to risk management. This paper defines agricultural economic resilience as the capacity of agricultural systems to maintain core functions and rapidly recover and reorganize after facing external disturbances such as natural disasters, market fluctuations, or policy shocks. This concept typically comprises two dimensions: risk resistance capability and recovery/reorganization capability. Risk-bearing capacity emphasizes pre-shock defensive mechanisms—such as resource reserves and structural redundancy—that shield systems from direct external disruption. Recovery and reorganization capacity focuses on post-shock self-repair, resource reallocation, and transition toward more adaptive pathways. Holling first defined resilience as an ecosystem’s capacity to return to its original state after disturbance. This concept was later extended to economics, emphasizing dynamic adaptation over static equilibrium [16]. In recent years, scholars have conceptualized agricultural economic resilience as a dynamic capability encompassing three dimensions: risk resistance, adaptive adjustment, and innovative reconstruction [17].

The cultivation of agricultural economic resilience is influenced by multiple factors, including crop diversity, supply chain stability, farmer adaptive capacity, and climate adaptation capabilities. (1) Crop Diversity. Diversifying crop types and varieties disperses the risk of single-crop failure and enhances overall output stability. Research indicates that diversified cropping systems demonstrate greater resilience to extreme weather events, as different crops exhibit varying sensitivities to climate and pests, creating complementary effects [18]. (2) Supply Chain Stability. Supply chain resilience depends on the redundancy of logistics networks, processing facilities, and market channels. Diversified procurement channels and localized processing facilities within the supply chain can maintain raw material supply and product circulation during external shocks, thereby sustaining agricultural economic operations [19]. (3) Farmer Adaptability. Farmers’ learning, innovation, and organizational capacities serve as critical micro-level drivers of resilience. Households equipped with technical training, information access, and cooperative networks are more likely to adopt new technologies, adjust cropping patterns, or transform business models, thereby enhancing the system’s adaptability and recovery speed [20]. (4) Climate Adaptation Capacity. Climate adaptation encompasses irrigation techniques, breeding of stress-tolerant varieties, and land management practices (e.g., water and nutrient retention). Literature indicates that agricultural systems with climate-adaptive management can rapidly recover yields after extreme weather events and achieve structural transformation over the long term, forming more resilient production patterns [7].

General economic resilience focuses on the recovery capacity of macroeconomic entities under macro-level shocks such as financial crises and industrial structural impacts, emphasizing industrial diversification, fiscal policy, and financial system flexibility [11]. Agricultural economic resilience, however, centers on the eco-economic coupling characteristics within agricultural systems, with its core lying in the dynamic regulation of natural capital such as land, climate, and biodiversity. Specific distinctions are reflected in: (1) Temporal scale. General economic resilience often focuses on medium- to long-term growth recovery; agricultural resilience emphasizes rapid recovery at seasonal and annual scales. (2) Spatial scale. Agricultural resilience is often analyzed at the county, watershed, or individual farm level, involving land use and local ecosystems; general economic resilience is typically examined at the provincial or national level. (3) Driving factors. Agricultural resilience is influenced by natural resources, technological innovation, and farmer behavior, emphasizing the maintenance of ecosystem services; general economic resilience relies more on financial, industrial policy, and labor market adjustments [21].

### 2.3 Theoretical analysis

#### 2.3.1 The direct impact of DID on the AER.

The DID, as a crucial lever of the Rural Revitalization Strategy (RRS), exerts a systematic driving effect on enhancing Agricultural Economic Resilience (AER) through institutional innovation and resource integration. AER emphasizes the capacity to resist, adapt, and innovate in response to natural risks, market fluctuations, and policy adjustments; meanwhile, DID reshapes the agricultural economic system via multiple dimensions and enhances the sustainability of its development capabilities [22].

First and foremost, DID significantly enhances the agricultural risk resilience capacity. Agricultural production has long been confronted with risks, including frequent natural disasters and volatile market prices. Coupled with inadequate rural infrastructure and backward information access, these risks further exacerbate operational hazards in agriculture [23]. The DID facilitates the return of talents with technological and financial advantages through initiatives such as financial support and tax incentives, and drives innovation in agricultural production practices [24–26]. Such initiatives have effectively strengthened the stability of agricultural production and fortified the capacity of the agricultural economy to withstand external shocks.

Secondly, DID facilitates the optimization of the adaptability and adjustment capacity of the agricultural system. According to Resource dependence theory, traditional agriculture is highly dependent on primary production factors such as land and labor, and lacks flexible adjustment mechanisms in response to risks. DID entails the construction of entrepreneurial incubation platforms, the organization of skill training, and the introduction of modern management practices, market operation models, and emerging technological elements to reconstruct the agricultural resource allocation system [27,28]. Such element integration and business model innovation empower the agricultural system to promptly adjust its production structure in response to market demand, thereby improving resource utilization efficiency and enhancing risk resilience capacity.

Finally, the DID effectively promotes the transformation and innovation capabilities of agriculture. Under policy guidance, entrepreneurs returning to their hometowns have become the core force of agricultural innovation by leveraging the technology, information, and networking resources accumulated in the city [29]. On the one hand, policies encourage industry university research cooperation, support entrepreneurs to connect with research institutions, accelerate the transformation of technological achievements such as new variety cultivation and smart agriculture, and enhance the technological content of agricultural production [30]. On the other hand, by creating entrepreneurial parks, industrial clusters, and other carriers, knowledge sharing and collaborative innovation among entrepreneurs can be promoted. This innovation driven development model not only promotes the extension of agriculture from traditional production to the entire industry chain, but also stimulates the endogenous growth momentum of the agricultural economy and enhances the system’s innovation resilience. Based on this, this paper puts forward a hypothesis:

H1: The DID can enhance the AER.

#### 2.3.2 Mechanism effect of farmers’ income (FAR).

Endogenous growth theory emphasizes the driving role of human capital accumulation and knowledge spillover on economic sustainability, which proposes that income growth not only reflects the improvement of resource allocation efficiency but also stimulates the investment capacity of households, forming a virtuous circle of “income - human capital - productivity” [31]. It proposes that income growth not only reflects the improvement of resource distribution efficiency but also forms a virtuous cycle of “income-human capital-productivity” by stimulating the investment capacity of households. The DID directly enhances the disposable resources of farm households by broadening business income and wage income. The increase in FAR increases their investment in human capital, such as education and skill training, and at the same time enhances their understanding and acceptance of modern agricultural technology [32] This synergistic upgrading of human and technological capital can systematically optimize agricultural production technology, thereby improving the agricultural system’s adaptive efficiency and recovery potential in response to external shocks.

At the same time, higher FAR increases farmers’ disposable capital, which they use for productive investment and consumption upgrading activities, such as purchasing agricultural machinery, improving soil and purchasing high-quality agricultural materials. This behavioral adjustment gradually spreads to the optimization of the regional economic structure [33,34]. For example, the DID has spawned local micro, small and medium-sized enterprises to form a synergistic network upstream and downstream of the industrial chain, enhancing the economic system’s self-repairing capacity. Moreover, the growth of FAR promotes AER by strengthening social liquidity capital. At the same time, income diversity reduces the transmission intensity of external shocks to the household economy, building a buffer mechanism for risk diversification [35]. In addition, higher-income accelerates the efficiency of technology diffusion, high-income farmers are more likely to bear the cost of trial and error of new technologies, and their successful experience can form a demonstration effect to promote the overall technological upgrading of the region, thus enhancing the adaptive adjustment capacity of agricultural production. Based on this, this paper puts forward the hypothesis:

H2: The DID can enhance the AER by increasing the FAR.

#### 2.3.3 Mechanism effect of rationalization of industrial structure (STR).

The theory of new structural economics emphasizes that the efficiency and stability of the economic system depend on the dynamic matching of industrial structure and factor endowment [31,36]. STR means that the allocation of production factors in agriculture and related fields becomes more coordinated, inefficient duplication of inputs is reduced, and an industrial pattern with a clear division of labor and complementary advantages is formed. Introducing modern management concepts, agricultural production from decentralization and homogenization to specialization, chain, and structural optimization can significantly improve the agricultural system’s resource utilization efficiency to cope with external shocks and create more adjustment space [37].

From an economic perspective, the indirect effects of STR manifest themselves on three levels. Returning entrepreneurs first deepen the connection between agriculture and the secondary and tertiary industries, establishing an efficient and coordinated industrial chain [38]. This integration reduces transaction costs by minimizing losses in the distribution chain, while significantly enhancing agriculture’s ability to respond to market fluctuations. When external shocks occur, upstream and downstream entities can flexibly adjust supply and demand through information sharing and feedback mechanisms based on close collaboration, thereby maintaining the basic functioning of the regional economy. Second, STR promotes specialized division of labor, effectively reducing resource misallocation [39]. Under the traditional small-scale farming model, farmers’ repetitive investment in similar production factors leads to low efficiency. Returnees guide farmers to focus on their comparative advantages based on their resource endowments through cooperatives or regional brand development, forming a differentiated production structure that avoids internal competition within the region and improves overall resource allocation efficiency [40]. Finally, a complete industrial chain creates the foundational conditions for technological penetration. The costs of developing and promoting new technologies are shared across all links in the industrial chain, lowering the barriers to adoption. Meanwhile, clear industrial scenarios make technology applications more targeted. This environment accelerates the large-scale implementation of technologies such as smart equipment and digital agriculture, systematically enhancing the adaptability and risk-resistance of agricultural production [41]. Based on this, this paper puts forward the hypothesis:

H3: The DID can enhance the AER by promoting the STR.

**Fig 2:**
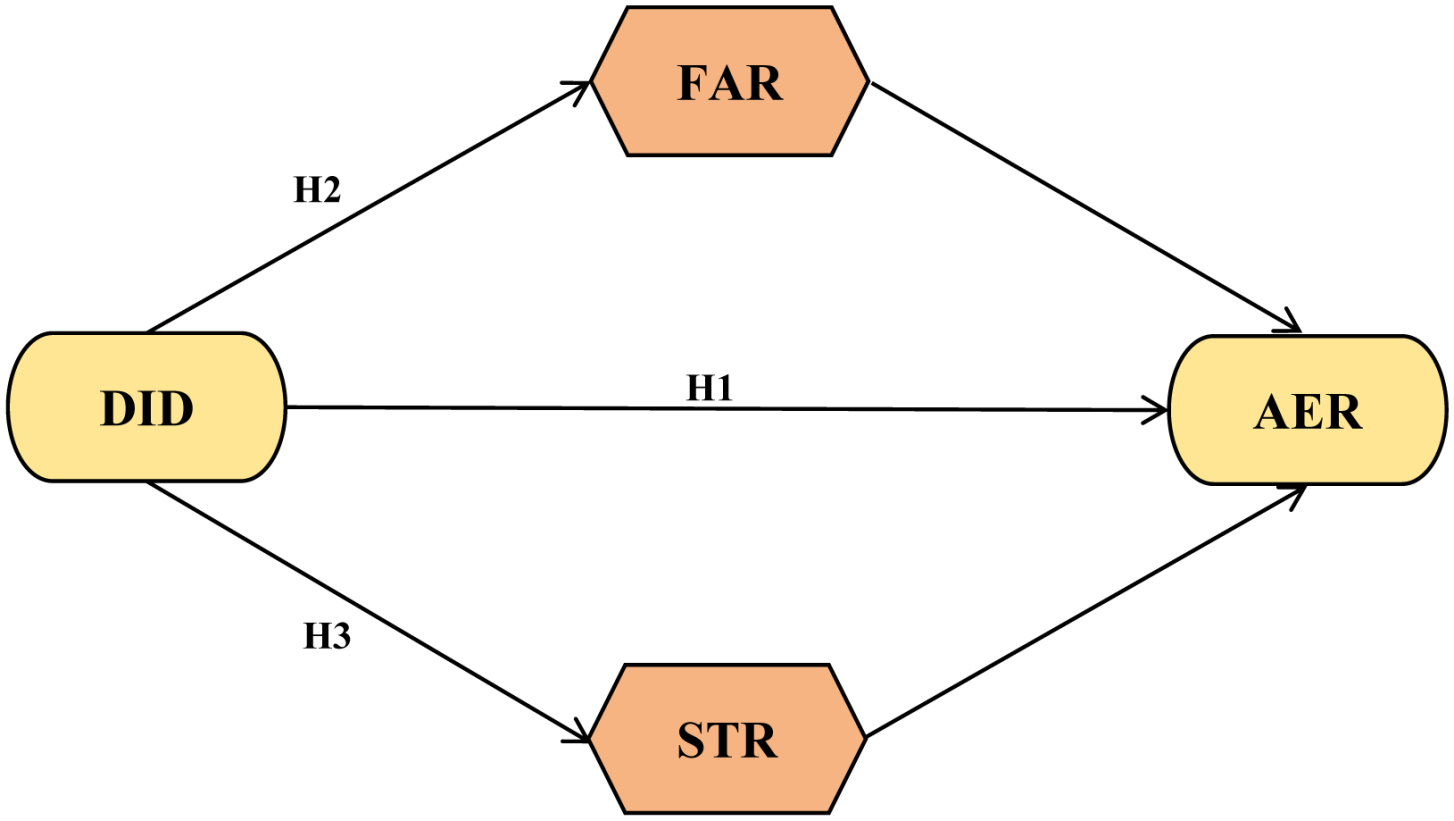
Mechanism diagram of the impact of DID on AER.

According to the theoretical analysis, this paper constructs a re search framework to analyze the impact mechanism of DID on AER(Fig 2).

## 3 Materials and methods

### 3.1 Data sources

From 2012 to 2022, this article collected county panel data from 2005 districts and counties in 30 provinces in China. Among them, the total number of pilot counties that returned to their hometowns for entrepreneurship was 243. In order to balance the panel data and shrink the control variables by 99%, the year when the policy was implemented and the subsequent years are set as treat period as 1, and the rest as 0. The set of pilot counties for hometown – return entrepreneurship in this research is sourced from announcements by the National Development and Reform Commission (NDRC). Data for the remaining variables are retrieved from the China County Statistical Yearbook and the China Statistical Yearbook. Table 3 presents the descriptive statistics of the key variables.

**Table 1:** System of indicators for evaluating the agricultural economic resilience.

Level 1 indicators	Level 2 indicators	Level 3 indicators	Expected impact	Calculation method
Risk resilience	Risk Absorption	Savings per capita	+	Savings balance at the end of the year/total population at the end of the year
		Food production per capita	+	Total food production/total population at the end of the year
		Number of employed people	+	Employees per unit at the end of the year
	Risk Resistance	Production capacity	+	GDP per capita
		Export dependence	–	Exports/Gross Domestic Product
		Industrialization	–	Secondary Industry/Gross Domestic Product
		Financial self-sufficiency	+	General budget revenue/general budget expenditure of local finances
Restoration of reorganization capacity	Economic recovery	Size of market investment	+	Total investment in fixed assets/total population at the end of the year
		Market Consumption	+	Total retail sales of consumer goods/total population at the end of the year
		Population urbanization rate	+	1-Rural population/Total population
		Medical coverage rate	+	Number of beds in hospitals and health centers per 10,000 people
		Sulfur dioxide emissions per unit of GDP	–	Sulfur Dioxide Emission/Gross Regional Product
	Innovation potential	Broadband access per 10,000 people	+	Regional broadband access/million people
		Number of fixed-line telephones per 10,000 people	+	Number of fixed-line telephones in the region/million people
		Number of students enrolled in general secondary schools per 10,000 people	+	Number of general secondary school students enrolled in school/million
		Value added of tertiary industry	+	Value added of tertiary industry in the region

**Table 2:** Indicator weighting factors.

Level 3 indicators	Information entropy value	Information utility value	Weighting factor
Savings per capita	0.98670	0.01330	6.408%
Food production per capita	0.97483	0.02517	12.125%
Number of employed people	0.97180	0.02820	13.582%
Production capacity	0.99283	0.00717	3.452%
Export dependence	0.99999	0.00001	0.005%
Industrialization	0.99944	0.00056	0.270%
Financial self-sufficiency	0.97812	0.02188	10.538%
Size of market investment	0.99565	0.00435	2.097%
Market Consumption	0.98515	0.01485	7.153%
Population urbanization rate	0.99999	0.00001	0.005%
Medical coverage rate	0.99229	0.00771	3.714%
Sulfur dioxide emissions per unit of GDP	0.99995	0.00005	0.024%
Broadband access per 10,000 people	0.97773	0.02227	10.725%
Number of fixed-line telephones per 10,000 people	0.98226	0.01774	8.547%
Number of students enrolled in general secondary schools per 10,000 people	0.99566	0.00434	2.090%
Value added of tertiary industry	0.96000	0.04000	19.266%

**Table 3:** Results of descriptive statistics.

Variables	N	Mean	SD	Min	Median	Max
AER	22055	0.045	0.022	0.015	0.039	0.348
DID	22055	0.073	0.260	0.000	0.000	1.000
STR	22055	0.385	0.184	0.000	0.378	0.988
FAR	22055	9.345	0.519	7.607	9.377	11.322
UIG	22055	2.312	1.178	0.022	2.068	15.518
AMH	22055	9.477	1.863	0.000	9.859	13.892
AIS	22055	1.125	1.242	0.000	0.869	65.099
ATL	22055	11.283	0.951	6.621	11.427	14.261

### 3.2 Variable definitions

Dependent variable: agricultural economic resilience (AER). The construction of this indicator system strictly follows the AER theoretical framework proposed by Martin (21), which divides resilience into two core dimensions: risk resistance capacity (including risk absorption and resistance) and recovery and restructuring capacity (including economic recovery and innovation potential). To precisely align with agricultural economic characteristics, we conducted targeted optimizations: In the risk absorption layer, we included “per capita grain production” to directly reflect food security, which is the foundation of agriculture; In the risk resistance layer, we introduced the “industrialization” (negative) indicator to capture the potential crowding-out effect of the secondary sector on agricultural resources, while using the “fiscal self-sufficiency rate” to measure the capacity of local governments to support agriculture; In the economic recovery layer, we adjusted the “market investment/consumption scale” per capita to align with the scale of rural economies and added the “medical coverage rate” to address shortcomings in rural public services; In the innovation potential layer, indicators such as “broadband access per 10,000 people” and “number of middle school students per 10,000 people” were selected to focus on the critical role of the digital divide and human capital in rural innovation. In summary, this system inherits the core of classical theory while highlighting the uniqueness of AER through agriculture-sensitive indicators (such as grain production, industrialization level, and digital infrastructure), combining theoretical rigor with practical explanatory power. Based on the authenticity and availability of data and science, this paper selects 16 third-level indicators to measure the development level of AER, as shown in Table 1.

The reason why risk resilience and recovery/reorganization capacity are set as primary indicators when measuring agricultural economic resilience is that these two dimensions comprehensively capture the dual dynamic characteristics of the system before and after shocks. Research indicates that resilience is essentially a composite process of“disturbance resistance, recovery, and transformation”, and a single dimension cannot fully reflect its entirety [26]. Risk resilience focuses on preemptive defense characteristics such as resource redundancy, structural diversity, and early warning mechanisms, serving as the key determinant of whether a shock leads to system failure. Recovery and restructuring capacity, meanwhile, centers on post-shock output recovery speed, resource reallocation efficiency, and the ability to transition toward more adaptive production models. Together, these two dimensions collectively determine the overall resilience level of the agricultural economy [24]. Furthermore, empirical analyses indicate that quantifying these two dimensions separately enhances the explanatory power and policy guidance of the indicator system. This enables policymakers to precisely identify “weak defense and slow recovery” segments, thereby formulating targeted support policies [39]. Consequently, the dual-layer framework centered on risk resilience and recovery-reorganization capacity aligns with the latest developments in resilience theory while offering strong operational feasibility and diagnostic value.

This study aims to employ the entropy – weighting approach to analyze the panel data of 2005 counties in China from 2012 to 2022. Subsequently, it will compute the weights and ultimately determine the level of AER. The entropy – weighting approach is adopted due to its objectivity, and the specific treatment process is as follows:

First, the data are standardized to determine the original data matrix and construct the initial matrix X.


$$\begin{array}{c} X={\left(x_{ij}\right)}_{n*m}=[\begin{array}{ccc} x_{\text{1}\text{1}} & \cdots & x_{\text{1}m}\\ \vdots & \ddots & \vdots \\ x_{n\text{1}} & \cdots & x_{nm} \end{array}] \end{array}$$ (1)

Where $X_{ij}$ represents the jth indicator of the ith sample (i=1,2,⋯,n,j−1,2,⋯,m), the dimensionless transformation of the data requires standardization of the data.

When the indicator is a positive indicator, the normalization formula is:


$$y_{ij}=\frac{x_{ij}-x_{min}}{x_{max}-x_{min}}$$ (2)

When the indicator is a negative indicator, the normalization formula is:


$$y_{ij}=\frac{x_{max}-x_{ij}}{x_{max}-x_{min}}$$ (3)

After the end of the indicator normalization process, the panning process is performed with the elimination of negative values:


$$\overset{\prime }{\underset{ij}{y}}=y_{ij}+d$$ (4)

Where d is the magnitude of the indicator shift, for the process of standardization of some of the indicator values, it will appear smaller values or negative values, through the standardization of the value of the shift process, to eliminate this phenomenon at the same time also make the calculation more uniform and convenient. In this study, because the values of some indicators are very close to each other, we chose a slightly larger shift amplitude, and the shift amplitude in this study is 0.001, and then we got the converted matrix $\overset{\prime }{\underset{ij}{y}}$.

Next, the weighting coefficients were calculated using the weighting method:


$$P_{ij}=\frac{\overset{\prime }{\underset{ij}{y}}}{\sum_{i=\text{1}}^{n}\overset{\prime }{\underset{ij}{y}}}$$ (5)

The entropy value is subsequently calculated:


$$e_{j}=\frac{\text{1}}{\text{ln}(n)}\sum_{i=\text{1}}^{n}P_{ij}\text{ln}\left(P_{ij}\right)$$ (6)

Where $e_{j}$ is the entropy value of the jth indicator.

Calculate the coefficient of variation:


$$g_{j}=\text{1}-e_{j}$$ (7)

Where $g_{j}$ is the coefficient of variation of the jth indicator, the larger the value of $g_{j}$, the higher the degree of the role of the indicator in the comprehensive evaluation index system.

Again, calculate the weight:


$$\omega_{j}=\frac{g_{j}}{\sum_{j=\text{1}}^{m}g_{j}}$$ (8)

Where j=1,2,⋯,m, this $\omega_{j}$ is the final weight coefficient of each indicator. Get the final weight system as shown in Table 2.

Independent variable: Regarding the pilot returning-home entrepreneurship policy (DID), for each county, DID is set to 1 in the year when it is designated as a pilot county and in subsequent years. In no other situation can it take a value other than 0, that is, it is set to 0 otherwise.

Mediating variables: Farmers’ income (FAR) and the rationalization of industrial structure (STR) are considered. To gauge FAR, this study aims to employ the statistical metric “per - capita disposable income of rural residents” from the China County Statistical Yearbook. As for STR, this paper suggests using the Theil index for measurement. When the Theil index approaches 0, it indicates that the output – value or income gap among various industries is narrower, signifying a more STR. Conversely, when the Theil index nears 1, it implies that the output – value or income gap among industries is more pronounced, meaning the industrial structure is less rational.

Control variables: Regarding relevant studies, the control variables incorporated in this research are as follows:① The urban - rural earnings gap (UIG), which is gauged by the quotient of the average disposable income of urban inhabitants and that of rural inhabitants.② Land availability (AMH), determined by calculating the machine – harvested area in each region.③ Agricultural technological sophistication (AIS), computed by dividing the total power of agricultural machinery in each region by the total population of that region.④ Industrial magnitude (ATL), ascertained based on the number of workers engaged in agriculture, forestry, animal husbandry, and fishery. There exists no alternative but to adopt these measurements for the sake of comprehensively accounting for various influencing factors in the research.

The variable definitions and descriptive statistics for the variables are detailed in Table 2 and Table 3, respectively.

### 3.3 Research methodology

(1) Difference-in-Differences model: This study employs the national county – level panel data from 2012 to 2022. It aims to apply the Difference-in-Differences model to explore the influence of the DID on the AER. The detailed model is structured as follows:

$${AER}_{it}=\alpha_{\text{0}}+\alpha_{\text{1}}DID_{it}+\sum \gamma \;control_{it}+\mu_{i}+v_{t}+\varepsilon_{it}$$ (9)

In the formula (9), ${AER}_{it}$ is the agricultural economic resilience, i and t denote the region and time respectively. $\alpha_{\text{0}}$ is the intercept term, DID is the pilot policy of returning to the countryside to start a business. $\alpha_{\text{1}}$ is the core variable of interest in this paper, denotes the impact of the pilot policy of returning home and entrepreneurship on the agricultural economic resilience. $control_{it}$is the set of all control variables that may affect the explanatory variables, and its regression coefficient is γ, $\mu_{i}$ and $v_{t}$ is the fixed effect of region and time, $\varepsilon_{it}$ is the random disturbance term.

(2) Mediation Effect model: To verify the mediating role of FAR and STR in the relationship between DID and AER, this paper references the mediating – effect testing protocol put forward by Wen and Ye [42]. There’s no option but to conduct analysis using the Bootstrap two – step technique. The specific formula is presented as follows:


$${Mediator}_{it}=\alpha_{\text{0}}+\phi_{\text{1}}DID_{it}+\sum \gamma \;{control}_{it}+\mu_{i}+v_{t}+\varepsilon_{it}$$ (10)

$${AER}_{it}=\alpha_{\text{0}}++\delta_{\text{1}}DID+\delta_{\text{2}}{Mediator}_{it}+\sum \gamma \;{Control}_{it}+\mu_{i}+v_{t}+\varepsilon_{it}$$ (11)

In the formula (10), (11) ${Mediator}_{it}$ is the mediating variable, including the NFR and the STR. The formula (10) is used to test whether the DID has an effect on the mediating variable, and the formula (11) is used to test whether the mediating variable has a mediating effect in the relationship between the DID and the AER. Other symbols have the same meaning as equation (1).

## 4 Results

### 4.1 Spatial and temporal characteristics of agricultural economic resilience

**Fig 3:**
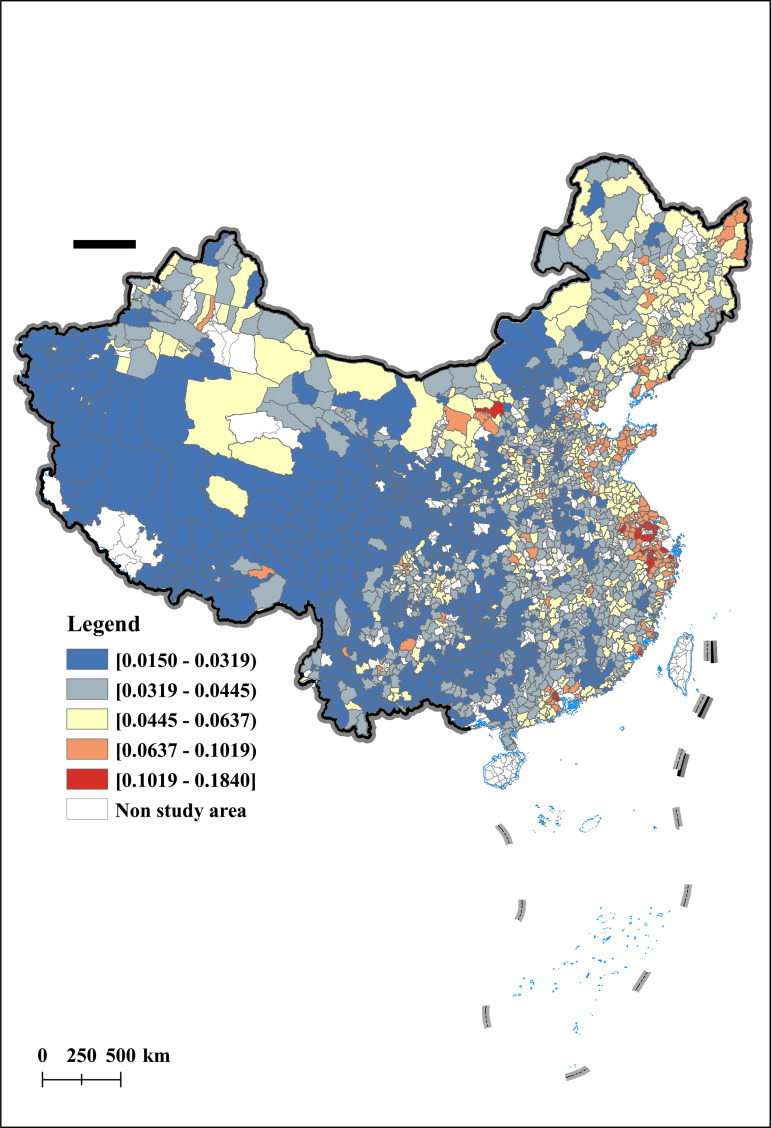
Spatial and temporal characteristics of agricultural economic resilience (Year:2012).

**Fig 4:**
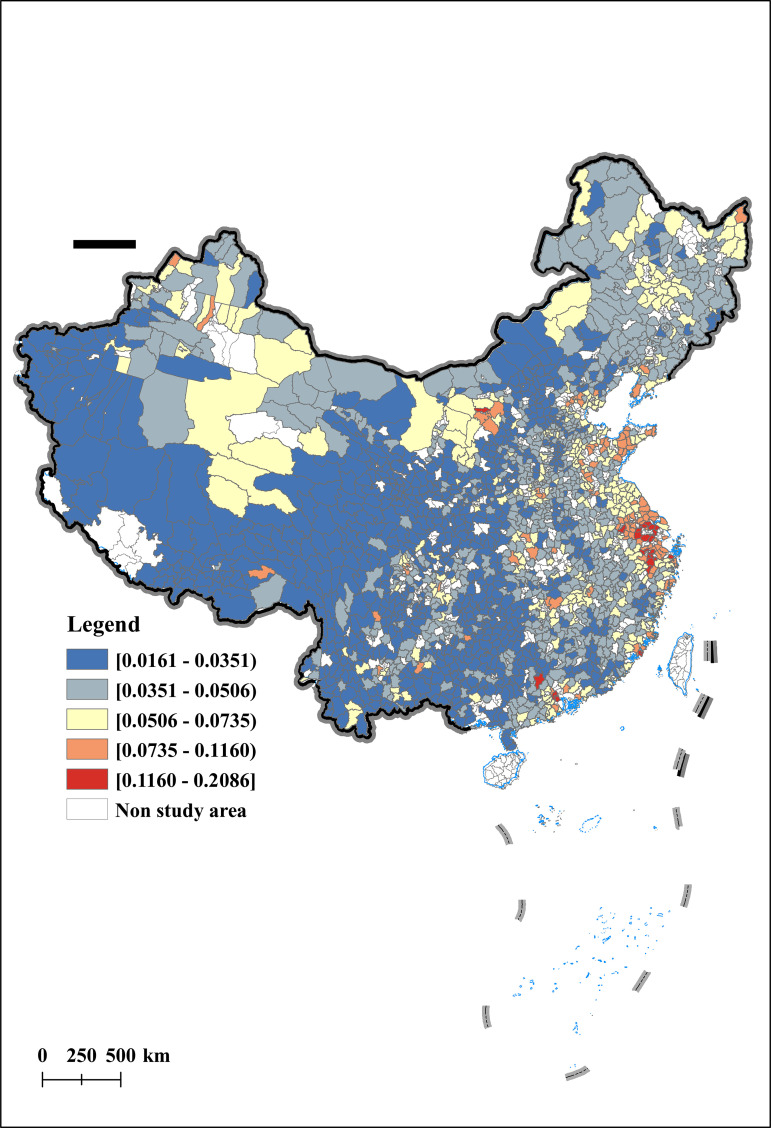
Spatial and temporal characteristics of agricultural economic resilience (Year:2015).

**Fig 5:**
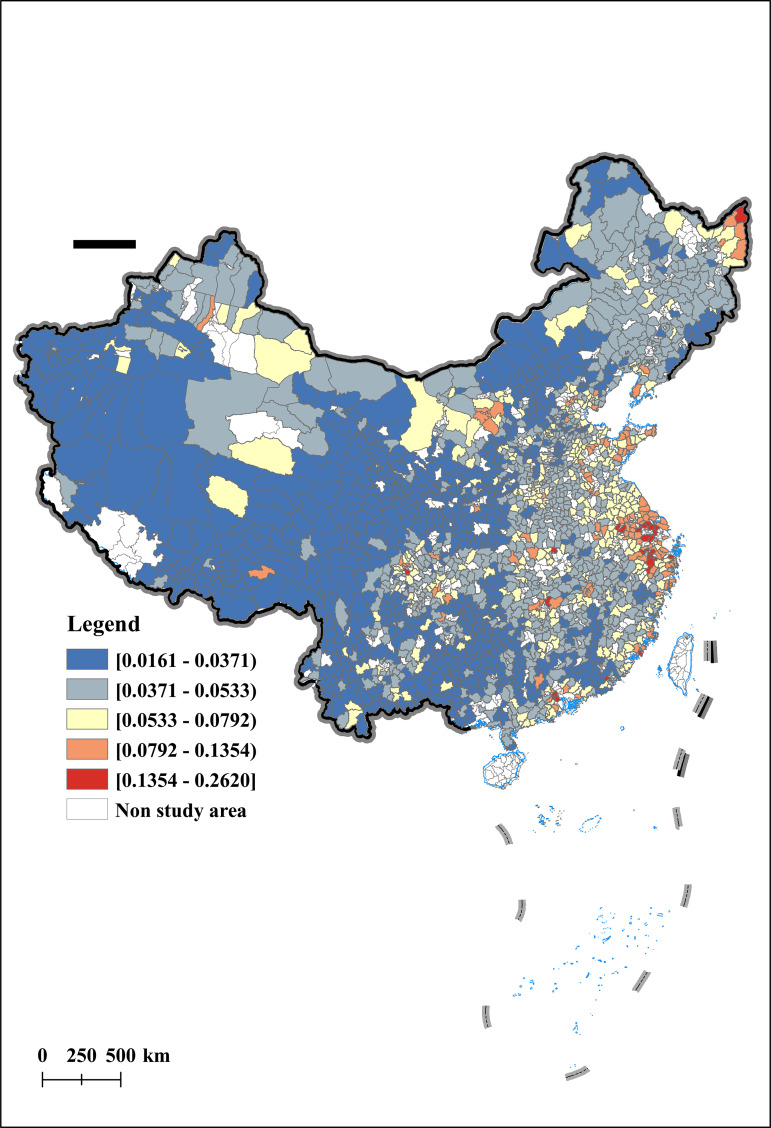
Spatial and temporal characteristics of agricultural economic resilience (Year:2019).

**Fig 6:**
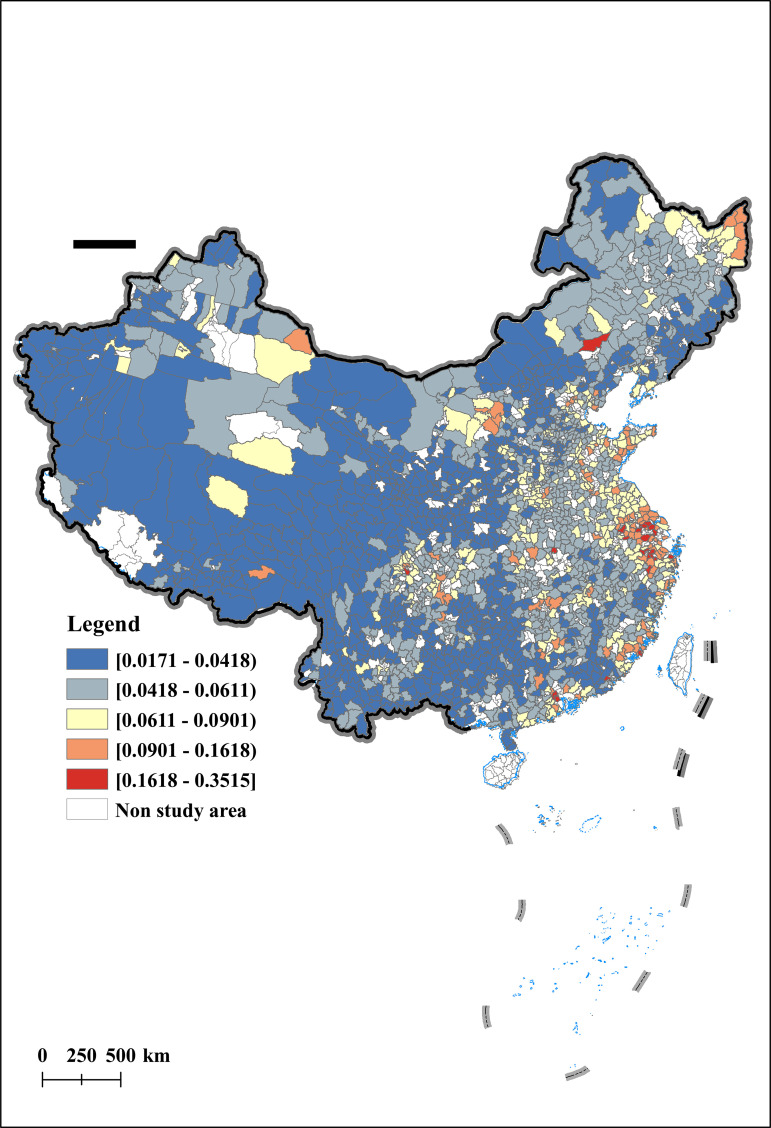
Spatial and temporal characteristics of agricultural economic resilience (Year:2022).

This article uses the Arc GIS technology platform to conduct spatial visualization analysis of AER in 2012, 2015, 2019, and 2022. The results are shown in Figs 3, 4, 5, 6. From a temporal perspective, the AER shows a stable and increasing trend, with an average value increasing from 0.0399 to 0.0510. From a spatial perspective, the AER exhibits significant spatial imbalances. Specifically, the AER is manifested in a spatial distribution pattern of high in the east and low in the west, and high in the north and low in the south. Among them, the AER in the Yellow River Delta and Yangtze River Delta has always been at a high level, which is consistent with the spatial pattern of China’s economic development. In addition, the AER of the Songjiang Plain has gradually taken the lead in Northeast China, forming a new highland for agricultural economic development, once again confirming the important position of the Songjiang Plain as one of China’s major grain producing areas. In contrast, under the influence of extreme natural disasters such as drought and sandstorms, the AER in northwest China is gradually decreasing.

To demonstrate once again that AER exhibits spatial imbalance characteristics, this paper analyzes the spatial autocorrelation of AER through global autocorrelation analysis using Moran’s I index in Geoda software. The results are shown in Table 4. From 2012 to 2022, the Global Moran’s I of AER showed a fluctuating downward trend, decreasing from 0.031 in 2012 to 0.027 in 2022, indicating a decrease in the spatial agglomeration intensity of AER. The maximum value of 0.032 appeared in 2020, which may be due to China’s achievement of the goal of comprehensive poverty alleviation in 2020. The infrastructure, industrial development, and public services in poverty-stricken areas have significantly improved. By filling in the gaps, the AER in rural areas has been enhanced, regional disparities have been narrowed, and the spatial correlation of AER between regions has been strengthened. Overall, both the global and local Moran indices are significantly positive above the 5% level, indicating that the spatial distribution of AER is not uniform or random, but presents significant spatial correlation.

**Table 4:** Global Moran’s I of agricultural economic resilience from 2012 to 2022.

Years	Global Moran’s I	Z	P
2012	0.031^**^	2.927	0.033
2013	0.031^**^	2.219	0.021
2014	0.030^**^	3.023	0.002
2015	0.030^***^	4.140	0.000
2016	0.029^***^	5.038	0.000
2017	0.029^**^	2.823	0.035
2018	0.030^**^	2.303	0.047
2019	0.030^**^	3.113	0.015
2020	0.032^***^	4.294	0.000
2021	0.028^***^	4.882	0.000
2022	0.027^***^	5.021	0.000

Notes: ^***^, ^**^ and ^*^ denote significance at the 1, 5 and 10 percent levels, respectively.

### 4.2 Preliminary test of the parallel trend hypothesis

When performing the difference – in – differences method test, the crucial aspect is that prior to policy implementation, there should be no substantial divergence in the time – trends of agricultural economic resilience between the pilot counties (treatment group) and non – pilot counties (control group). Instead, they should exhibit approximately similar change trends. Only after policy implementation can significant differentiation occur.

**Fig 7:**
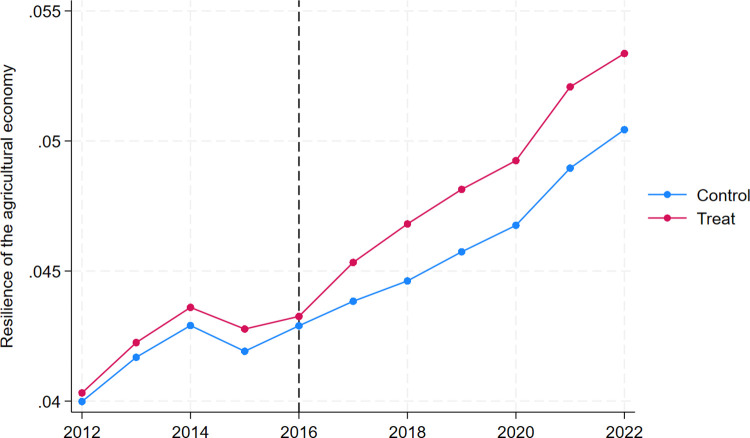
Parallel trend test for Difference-in-Differences model.

**Fig 8:**
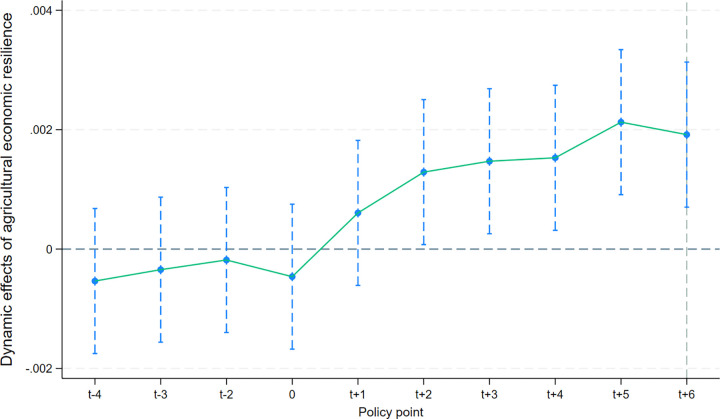
Parallel trend test for Difference-in-Differences model.

Figs 7 and 8 together reveal this situation, according to which it is found that the agricultural economic resilience of the regions had the same change profile without significant differences before the regions were selected for the pilot list of returnee entrepreneurs. In contrast, after 2016, i.e., after the regions were selected for the pilot list of returnee entrepreneurs, significant differences emerged, as shown by the fact that the AER of the pilot counties and the non-pilot counties together showed an increasing trend. However, the growth of AER in the treatment group is higher than in the control group.

According to Fig 8, the dynamic effect of each time point before 2016 is not significant. The dynamic, positive effect of AER is significant at the t + 2 time point in 2018, the 95% CIs are higher than 0. This indicates that there is a lag in the impact of policies on AER. The possible reason is that, on the one hand, from the introduction of national policies to the specific implementation in counties and villages, it takes an average of 6–12 months to decompose and refine the supporting measures, resulting in the inability to immediately release policy dividends; On the other hand, agricultural production requires a cultivation cycle, which directly leads to policy interventions that cannot be immediately transformed into production results, forming a “time difference” in policy responses. It is worth noting that in the fifth year of policy implementation (2021), its positive impact on AER reached its peak. The fundamental reason for this phenomenon is that in 2021, the number of Chinese entrepreneurs returning home reached 11.2 million, achieving a historic breakthrough and directly providing labor security and technical support for agricultural production. Consequently, this study provisionally infers that around 2016, compared to non – pilot counties, the pilot policy of rural – return entrepreneurship led to a remarkable elevation in AER within pilot counties. There’s no doubt that the policy exerts an impact on AER.

### 4.3 Policy effect analysis based on difference-in-differences model

Table 5 clarifies the regression results of the policy effect analysis based on the Difference-in-Differences model, this time using the DID double difference model for the analysis, with AER as the explanatory variable, the cross-multiplier term of the pilot districts and counties treated and the period of the policy implementation period constitutes the DID variable as the explanatory variable, and the urban-rural income gap (UIG), land endowment (AMH), agricultural technology level (AIS), and industrial scale (ATL) as control variables, and regression analysis considering individual differences in districts and counties and year differences. The results are as follows:

**Table 5:** Benchmark regression results.

Variables	M (1)	M (2)
	AER	AER
DID	0.0017^***^	0.0016^***^
	(6.178)	(6.020)
UIG		−0.0002^**^
		(−2.100)
AMH		−0.0009^***^
		(−8.636)
AIS		0.0009^***^
		(11.804)
ATL		−0.0075^***^
		(−12.141)
Cons	0.0400^***^	0.1327^***^
	(277.460)	(18.789)
Year	control	control
Prov	control	control
N	22055	22055
R^2^	0.210	0.224
Adj. R^2^	0.131	0.145

Notes: The values in parentheses are t-values. ^***^, ^**^ and ^*^ denote significance at the 1, 5 and 10 percent levels, respectively.

In the baseline regression analysis M (1), solely the DID variable is included. The coefficient of this variable amounts to 0.0017 and is statistically significant at the 1% significance level. In other words, relative to non – pilot provinces, the implementation of the DID has raised the AER of pilot counties by 0.17%. In M (2), four control variables are incorporated on the basis of M (1). The regression coefficient of DID stands at 0.0016, and there’s no way it can be anything but significant at the 1% significance level. Hypothesis H1 is initially validated to demonstrate that.

It is worth noting that, despite DID having a significant positive impact on AER, the effect is relatively small. The reason for this phenomenon is as follows: First, the core dependent variable of this study, “economic resilience,” is a composite index constructed through standardization and weighting of multiple basic indicators. Due to significant differences among regions in the original indicators constituting this index, and the synthesis process itself tending to compress the final score within a relatively limited numerical range, the overall numerical scale of the economic resilience variable is generally low. Second, the key independent variable DID is a binary dummy variable (0 = control group, 1 = treatment group), and the intuitive economic meaning of its regression coefficient is the average absolute difference in economic resilience scores between the treatment group and the control group. Precisely because the numerical range of the dependent variable itself is small, even if the treatment effect is statistically significant and practically meaningful, its absolute numerical value reflected in the regression coefficient will naturally appear relatively small. Therefore, the small coefficient is a reasonable result of the combined effects of the small scale of the dependent variable and the binary dummy variable, and does not necessarily imply a weak treatment effect.

### 4.4 Robustness tests

#### 4.4.1 Placebo test.

The total number of districts in this sample is 2005. There are 243 pilot districts, so 243 cities are randomly selected from the 2005 districts, and these 243 cities are set as the new sample of false pilot districts. The rest of the districts are set as new as the control group to get the new subgrouping variable treat, which is then set as the cross-multiplier term with the period of the implementation of the policy, 2016 Period, and is set as the new DID, regress it according to the two-way fixed double difference model to get the interaction term coefficient and p-value, repeat this process 1000 times to get 1000 false pilot counties and DID, regress it according to the model, and finally get 1000 estimated coefficients and p-values, and make a kernel.

**Fig 9:**
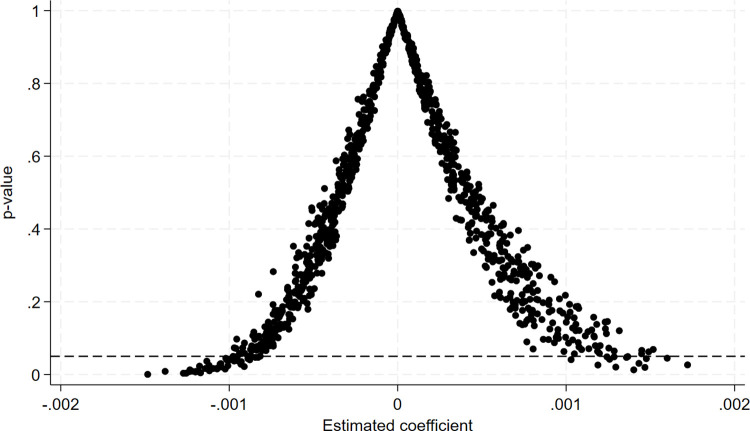
Kernel Density of Estimated Coefficients and p-value Distributi*on.*

Fig 9 shows that most of the 1000 regression coefficients Coefficient are concentrated near the 0 points, indicating that the AER of the treatment and control groups showed no significant difference after 2016. The baseline regression yielded the regression coefficient of the DID of 0.0016, which is an obvious outlier when compared with the results of the placebo test, the value of which belongs to the apparent outliers, and the black dots are the p-value, the black horizontal dashed line is the line with a p of 0.1, and most of the p-values in the results are more significant than 0.05, indicating that most of the coefficients are not significant. Again, this indicates that the DID caused a significant difference in the AER of the district and county between the treatment and control groups after 2016.

#### 4.4.2 PSM-DID analysis.

To further assess the reliability of the above benchmark findings, the Propensity Score Matching Double Difference (PSM – DID) model is adopted for robustness checks. The dataset in this study encompasses 2005 districts and counties. However, only 243 districts and counties belong to the treatment group, while the remainder constitute the control group. Given the substantial disparity in sample sizes between the two groups, Propensity Score Matching (PSM) is employed for sample filtering. PSM aims to identify a control group with characteristics similar to those of the treatment group. By doing so, it mitigates the influence of sample – selection bias on the estimation of policy impacts and ensures the comparability of the treatment and control groups prior to policy implementation. The logit regression approach is utilized to conduct nearest – neighbor matching with a 1:1 caliper and sampling with replacement. After implementing PSM, an appropriate sample of 5346 observations is secured.

**Fig 10:**
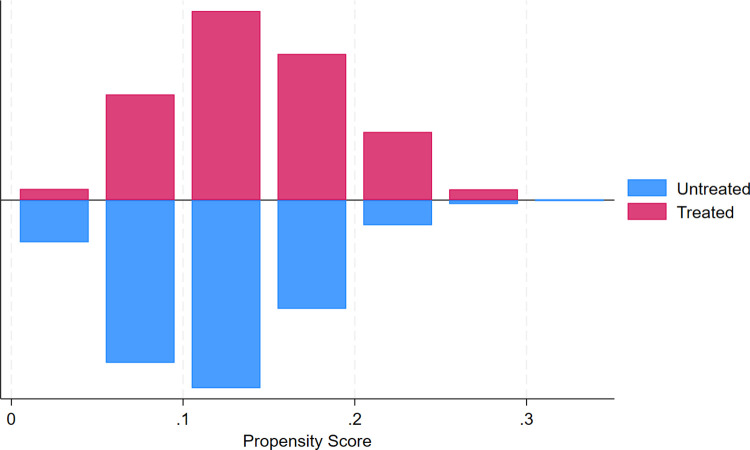
The corresponding balance test.

First of all, this propensity score matching (PSM) to balance the test, the results are shown in Table 6: urban-rural income gap (UIG), land endowment (AMH), agricultural technology level (AIS), industrial scale (ATL) as covariates involved in the matching, in which the urban-rural income gap (UIG), land endowment (AMH), industrial scale (ATL) in the matching of the pre-test difference between the two means of the two groups, the t-test difference is significant and the t-test difference is significant (p < 0.05) after the matching is performed, the difference between the two groups is significantly reduced, and it can be observed that the t-test test of the four covariates after the matching is not significant, and the deviations are all reduced to less than 5%, which indicates that there is a significant gap between the covariates of the treatment group and the control group in the original data. After PSM, the remaining two groups of samples are no longer different, indicating the necessity of conducting PSM. The above conclusion is further illustrated by Fig 10, where the change in the deviation of the covariates of the two groups of samples before and after PSM can be more intuitively observed. After matching, the kernel densities of the propensity score values of the treatment and control groups have been very close to each other, indicating that the matching is more effective and in line with the standard value assumption.

**Table 6:** Results of propensity score matching.

Variables	Unmatched	Mean		%bias	t-test	
	Matched	Treated	Control		t	p > t
UIG	U	2.254	2.320	−6.300	−2.730	0.006
	M	2.254	2.259	−0.500	−0.220	0.822
AMH	U	10.089	9.392	39.500	18.260	<0.001
	M	10.089	10.094	−0.300	−0.130	0.898
AIS	U	1.119	1.126	−0.600	−0.260	0.795
	M	1.119	1.138	−1.600	−0.670	0.501
ATL	U	11.593	11.240	40.300	18.100	<0.001
	M	11.593	11.589	0.400	0.160	0.871

Table 7 presents the regression results of the PSM – DID model. After applying propensity score matching (PSM), when control variables are not incorporated, the regression coefficient of DID is 0.0064, and it’s statistically significant at the 1% level. When control variables are included, the regression coefficient of DID amounts to 0.0013, again significant at the 1% statistical threshold. These results align closely with the benchmark findings. Evidently, this further validates the reliability of the regression results obtained from the benchmark model.

**Table 7:** PSM-DID robustness tests.

Variables	M (1)	M (2)
	AER	AER
DID	0.0064^***^	0.0013^***^
	(22.445)	(3.269)
UIG		−0.0008^**^
		(−2.155)
AMH		−0.0008^***^
		(−3.029)
AIS		0.0020^***^
		(9.260)
ATL		−0.0116^***^
		(−6.416)
Cons	0.0436^***^	0.1828^***^
	(336.256)	(8.698)
Year	YES	YES
Prov	YES	YES
N	5346	5346
R^2^	0.117	0.278
Adj. R^2^	0.106	0.264

Notes: The values in parentheses are t-values. ^***^, ^**^ and ^*^ denote significance at the 1, 5 and 10 percent levels, respectively.

#### 4.4.3 Other robustness tests.

(1) Excluding municipalities and counties. Municipalities under the central government are unique in their policy implementation paths due to their higher administrative hierarchy and resource allocation advantages. These regions are usually prioritized to take over the policy pilot tasks and rely on exceptional financial support to strengthen the integration of urban and rural factors. As the agricultural economy in this region accounts for a relatively low proportion, the policy focuses more on industrial linkage and the construction of urban-rural cooperative development mechanisms rather than strengthening the stability of the traditional agricultural production system. Consequently, the study employs the approach of excluding municipalities and counties to restructure the sample and validate the robustness of the benchmark regression outcomes. Table 8, M (1) displays the results of re – running the benchmark model. Here, the regression coefficient of the DID is 0.0012, with statistical significance at the 1% level. This indicates that the DID continues to have an impact on the AER.
(2) Excluding Provincial Directly Administered Districts and Counties. Provinces directly under the control of counties form an efficient policy implementation network through the vertical administrative system and can quickly superimpose multi-level support measures. Such regions are deeply embedded in the agricultural and industrialization systems, and the policy effectiveness mainly relies on the synergistic effect of industrial clusters, which emphasizes systematic resource integration rather than decentralized internal adjustment of agriculture. Similarly, the method of restructuring the sample by removing the counties directly under provincial jurisdiction is adopted to confirm the robustness of the benchmark regression results. The regression findings are presented in M (2) of Table 8. After excluding these provincial – directly – administered counties, the regression coefficient of the DID reaches 0.0017. Significance at the 1% statistical level further attests to the robustness of the benchmark regression results.
(3) Adjust the sample interval. Considering that large-scale public health events may significantly interfere with the action path of the pilot policy of returning to the hometown to start a business, this paper selects the local samples between 2012 and 2010. It regresses the benchmark model again, and M (3) in Table 8 further verifies the conclusion that the DID can affect the resilience of the AER.

**Table 8:** Other robustness tests.

Variables	M (1)	M (2)	M (3)
	AER	AER	AER
DID	0.0012^***^	0.0017^***^	0.0013^***^
	(4.378)	(6.403)	(5.188)
UIG	−0.0003^***^	−0.0002^*^	−0.0001
	(−2.614)	(−1.920)	(−0.830)
AMH	−0.0008^***^	−0.0008^***^	−0.0006^***^
	(−8.280)	(−7.962)	(−6.504)
AIS	0.0009^***^	0.0009^***^	0.0009^***^
	(11.983)	(12.084)	(9.539)
ATL	−0.0071^***^	−0.0073^***^	−0.0063^***^
	(−11.499)	(−11.903)	(−10.222)
Cons	0.1279^***^	0.1296^***^	0.1164^***^
	(18.095)	(18.431)	(16.543)
Year	YES	YES	YES
Prov	YES	YES	YES
N	21648	21626	18045
R^2^	0.217	0.222	0.154
Adj. R^2^	0.138	0.144	0.047

Notes: The values in parentheses are t-values. ^***^, ^**^ and ^*^ denote significance at the 1, 5 and 10 percent levels, respectively.

### 4.5 Heterogeneity analysis

Recognizing the substantial impact of locational factors on regional development, this research classifies the sample counties into three major geographical zones: the eastern, central, and western regions. Considering the variance in resource endowment, policy focus, and developmental phase across different areas, an interaction term between the explanatory variables and a dummy variable for county location is constructed to empirically explore the spatial heterogeneity of the relationship between the DID and the AER (see Table 9). As presented in Table 8, in the central and western regions, the regression coefficients of the DID interaction term are 0.0010 and 0.0027 respectively. Both coefficients are statistically significant at the 1% level. This suggests that the DID effectively boosts the resilience of the AER in the central and western regions, with a more pronounced effect in the western region compared to the central region. In contrast, the regression coefficients of the policy interaction term in the eastern region fail to meet the significance test requirements.

**Table 9:** Results of regional heterogeneity analysis.

Variables	East	Central	West
	AER	AER	AER
DID	0.0001	0.0010^***^	0.0027^***^
	(0.082)	(2.640)	(7.778)
UIG	0.0049^***^	−0.0010^***^	−0.0005^***^
	(8.357)	(−3.477)	(−5.244)
AMH	−0.0013^***^	−0.0011^***^	−0.0004^***^
	(−4.804)	(−5.303)	(−3.582)
AIS	0.0032^***^	0.0026^***^	0.0006^***^
	(9.218)	(11.939)	(9.621)
ATL	−0.0093^***^	−0.0056^***^	−0.0057^***^
	(−5.549)	(−5.710)	(−7.653)
Cons	0.1583^***^	0.1156^***^	0.0994^***^
	(8.058)	(10.063)	(11.912)
Year	YES	YES	YES
Prov	YES	YES	YES
N	21648	21626	18045
R^2^	0.217	0.222	0.154
Adj. R^2^	0.138	0.144	0.047

Notes: The values in parentheses are t-values. ^***^, ^**^ and ^*^ denote significance at the 1, 5 and 10 percent levels, respectively.

The reason may be that the agricultural industry chain in the central and western regions is shorter, the labor force outflow is severe, and the risk-resistant ability is weaker. The DID can bring capital, technology and management experience, quickly make up for the short board of the industrial chain, promote the integration of agriculture and secondary and tertiary industries, and form a more complete production system. Moreover, this “make up the short board” effect is especially obvious in the western region. The western region has a weak industrial base, but its ecological resources are rich but underdeveloped, so the policy effect is more prominent; the returnees use the characteristic resources, such as plateau agriculture, ethnic culture and innovative business model, to activate the idle elements, to enhance the efficiency of the space is more excellent. The eastern region, however, has a higher level of agricultural modernization and a mature industrial chain, so there is limited room for improvement brought about by the policy; at the same time, there are many local non-agricultural employment opportunities, and farmers’ entrepreneurial motivation is relatively insufficient, which has led to the policy effect not being manifested.

### 4.6 Mechanism analysis

The previous analysis shows that the DID does have a significant role in enhancing the resilience of the AER. To further identify the mechanism of the DID’s impact on the AER, this paper adopts the mediation effect model and selects the FAR and the STR to test the mechanism.

#### 4.6.1 Mediation mechanism test results of FAR.

The DID encourages farmers to return to their hometowns to start their businesses, introduces technology, capital and innovation elements, and promotes the extension of the agricultural industry chain and the integration of business forms. Entrepreneurial activities directly create local jobs and broaden farmers’ business and wage income channels. The FAR enhancement strengthens farmers’ ability to pay for technology adoption, risk resistance and asset investment and promotes the optimization and reorganization of production factors. At the same time, income growth activates rural economic vitality through the consumption multiplier effect, forming a diversified industrial structure and ultimately strengthening the adaptive capacity and resilience of the agricultural system to external shocks.

M (1) in Table 10 shows that the DID can significantly improve the FAR, and its regression coefficient is 0.0075. It is significant at a 1% statistical level, which indicates that the DID directly impacts the improvement of the FAR. On this basis, the dummy variable of the DID and the FAR are introduced into the same model for regression, and the results are shown in Table 10, M (2); the regression coefficients of the DID and the FAR for the AER are both significantly positive at the 1% level, which indicates that the DID can improve the FAR as an indirect effect of the role of the AER. H2 of this paper is supported.

**Table 10:** Results of the mediation effect test.

Variables	M (1)	M (2)	M (3)	M (4)
	FAR	AER	STR	AER
DID	0.0075^**^	0.0016^***^	−0.0137^***^	0.0016^***^
	(2.347)	(5.907)	(−3.580)	(5.966)
Theil				−0.0010^**^
				(−2.071)
NFR		0.0043^***^		
		(7.248)		
UIG	−0.1932^***^	0.0006^***^	0.0008	−0.0002^**^
	(−146.413)	(3.749)	(0.500)	(−2.093)
AMH	0.0036^***^	−0.0009^***^	−0.0014	−0.0009^***^
	(3.065)	(−8.802)	(−1.025)	(−8.651)
AIS	0.0027^***^	0.0009^***^	−0.0072^***^	0.0009^***^
	(3.061)	(11.660)	(−6.840)	(11.691)
ATL	−0.0317^***^	−0.0074^***^	−0.0372^***^	−0.0076^***^
	(−4.328)	(−11.930)	(−4.254)	(−12.199)
Cons	9.6993^***^	0.0908^***^	0.7989^***^	0.1335^***^
	(115.973)	(9.959)	(8.005)	(18.878)
Year	YES	YES	YES	YES
Prov	YES	YES	YES	YES
N	22055	22055	22055	22055
R^2^	0.941	0.226	0.666	0.224
Adj. R^2^	0.935	0.148	0.633	0.146

Notes: The values in parentheses are t-values. ^***^, ^**^ and ^*^ denote significance at the 1, 5 and 10 percent levels, respectively.

#### 4.6.2 Mediation mechanism test results of STR.

Table 10, M (3) reveals that the regression coefficient of the DID with respect to the STR exhibits a significant negative value. This implies that the implementation of this policy contributes to the improvement of the STR. Building on this, both the DID and the STR dummy variable are incorporated into the same regression model. The outcomes are depicted in M (4) of Table 10. Here, the coefficients of the DID dummy variable and the STR in relation to the AER are both significantly positive at the 1% level. This finding indicates that the STR serves as an intermediary in the process through which the DID enhances the AER, thereby validating Hypothesis H3 of this study.

The DID serves as an impetus for farmers to acquire new technologies and advanced management expertise. As a result, it facilitates the reallocation of agricultural resources and the vertical integration of the industrial chain. Entrepreneurs drive the agglomeration of production factors towards high – value – added segments, along with the integration of the three industries and specialized labor divisions. Additionally, the STR mitigates homogeneous competition and boosts total factor productivity. Moreover, the STR fortifies the resilience of the regional economic network via the technology diffusion effect. It creates a diversified and complementary industrial ecosystem, thereby enhancing the agricultural system’s adaptability and recovery efficiency when confronted with external shocks.

## 5 Discussion

Returnee entrepreneurship constitutes a highly distinctive and context-specific phenomenon of population mobility and employment in the PRC, characterized primarily by three core dimensions. First, it involves population flow from developed regions to less developed rural areas. Internationally, population migration is predominantly unidirectional toward developed regions; in contrast, China’s returnee entrepreneurship exhibits a two-way population flow pattern between urban and rural areas. Second, it emphasizes return to the place of origin (hometown). The selection of destinations for China-specific returnee entrepreneurship is not determined by a region’s entrepreneurial advantages, but rather by the returnees’ place of origin. Third, the selection of entrepreneurial industries is grounded in local resource endowments. Internationally, a common feature of returnee entrepreneurship is its focus on high-tech industries, with an emphasis on leveraging cutting-edge technologies acquired by returnees during their stay in developed countries. By contrast, the industry selection for China-specific returnee entrepreneurship is largely based on the resource endowments of rural areas, with the involved industries generally not classified as high-end [43]. In the field of returnee entrepreneurship research, a large body of literature has confirmed that returnees accelerate the formation of economic agglomeration, create employment opportunities, reshape regional industrial structures, and drive regional economic development through their entrepreneurial activities [44,45].

Although our research findings are consistent with existing studies that suggest that the DID is a catalyst for regional economic growth, can increase income, and promote industrial rationalization [46,47], there is little literature that delves into the relationship between returning home for entrepreneurship and rural agriculture, which clearly does not align with the original intention of the DID. The phenomenon of large-scale entrepreneurship with Chinese characteristics returning home is unparalleled in the world today. Its implementation purpose is to attract farmers to return to rural areas, build rural areas, and especially promote the modernization of agriculture. Therefore, this article attempts to incorporate DID and AER into the same analytical framework, to explore in depth the impact of DID on AER, and to analyze the specific impact mechanisms. Empirical research results indicate that the DID can significantly enhance the AER. Among them, FAR and STR play a positive role in enhancing the AER through DID. This research conclusion enriches the existing research findings that DID not only has economic development effects and employment effects for residents [48,49], but also can promote sustainable agricultural development.

## 6 Conclusions and recommendations

### 6.1 Conclusions

Employing the Difference-in-Differences (DID) model and the Mediation Effect (ME) model, this study conducts an empirical analysis using county-level panel data from the PRC spanning 2012–2022, aiming to explore the impact of DID on AER, as well as the mediating mechanisms of FAR and STR. The findings indicate that DID exerts a significant positive effect on enhancing AER, with distinct regional heterogeneity in the manifestation of this effect. The key findings are as follows: (1) AER presents a steady upward trend and exhibits significant spatial disparity, characterized by a spatial distribution pattern of “high in the east, low in the west, high in the north, and low in the south”. (2) On average, the implementation of the DID policy increases AER in pilot counties by 0.16 to 0.17 percentage points. This conclusion is validated by a series of robustness tests, including the placebo test, PSM-DID approach, and sample sensitivity analysis. (3) From a spatial dimension, the effect of DID is particularly pronounced in the central and western regions of the PRC. In contrast, the effect is statistically insignificant in the eastern region. (4) Further mechanism tests reveal that the effect of DID is transmitted through two mediating channels: FAR and STR. The improvement of FAR facilitates farmers’ adoption of new technologies and enhances their risk resilience capacity, thereby indirectly improving AER. Meanwhile, STR indirectly enhances AER by optimizing factor allocation efficiency and extending the industrial chain.

### 6.2 Recommendations

(1) Optimizing the Implementation of the DID: Drawing on Chinese county – level data from 2012 to 2022, this study applies the Difference-in-Differences model and formulates differentiated policy support frameworks to empirically analyze the influence of the DID on the AER, along with the impact mechanisms of FAR and STR. Considering the central and western regions’ weak industrial chains and the severe situation of factor outflow, it is essential to intensify infrastructure support and direct policy resources towards these areas. Special attention should be given to the development of cold – chain logistics, e-commerce platforms, and other industrial support systems. This will help to reduce the circulation radius of production factors. For the eastern region, the policy focus should be shifted to technology empowerment and model innovation, relying on digital technology to promote the intelligent transformation of the entire agricultural industry chain and to build a closed-loop ecology of “technology research and development - results transformation - commercial application”. The selection mechanism of pilot counties needs to be further improved by establishing a three-dimensional assessment model of “resource endowment-industrial foundation-policy demand”, prioritizing counties with rich ecological resources but underdeveloped.
(2) Focusing on the dual drivers for AER: On the one hand, STR should be core to promote the deep integration of the three industries through financial subsidies to guide social capital to invest in the deep processing of agricultural products, leisure agriculture and other chain extension projects, and cultivate industrial clusters with regional characteristics. Supporting leading enterprises and professional cooperatives to establish a “risk-sharing, benefit-sharing” mechanism of linking farmers and bringing them together to enhance the efficiency of industrial chain synergy and risk-resistant ability. On the other hand, it is necessary to improve the mechanism of FAR, relying on vocational colleges and scientific research institutions to build an integrated service platform of “technical training, business incubation and market docking”, focusing on the development of skills training such as digital agriculture and brand operation, to enhance the adaptive capacity of farmers to cope with market fluctuations.
(3) Establishing the return entrepreneurship ecosystem: The government should cultivate the market demand for new agricultural businesses through consumer subsidies, green certification and other policy tools, focusing on supporting organic agriculture, customized agriculture and other high-quality product supplies to create a differentiated market space for entrepreneurs returning to their hometowns. Establishing a county entrepreneurship information-sharing platform, dynamically releasing industrial chain gaps, technology needs and policy lists, and guiding entrepreneurial projects to dock with regional industrial planning accurately. In terms of improving farmers’ ability, building a “universal + customized” entrepreneurial support system is necessary, providing gradient training courses and financial products for different stages of the start-up and growth periods.

### 6.3 Limitations and future directions

This article analyzes the impact and mechanism of DID on AER from an economic perspective. However, DID has brought changes to rural areas that go far beyond the economic level, such as improving the happiness, sense of achievement, and sense of belonging of rural residents. The next step of research should focus on examining the structure and mechanism of the impact of DID on non economic development in rural areas, in order to prepare in advance for possible changes in rural economy, culture, society, ecology, and other aspects. Meanwhile, this study is based on the empirical results and mechanisms of Chinese rural families, particularly in accordance with China’s policy of returning home for entrepreneurship. Future research can conduct international comparative studies to explore the relationship between returning home entrepreneurship and agricultural economic resilience under different cultural backgrounds.

## Supporting information

S1 File Data. (XLSX)
